# Association between HIV duration and symptom distress among middle-aged and elderly people with HIV-infected in China: a cross-sectional study

**DOI:** 10.1186/s12877-022-03411-x

**Published:** 2022-09-05

**Authors:** Meilian Xie, Aiping Wang, Kerong Wang, Yanping Yu, Zhaoxia Lin

**Affiliations:** 1 Department of Public Service, The First Affiliated Hospital of China Medical University, Shenyang, Liaoning Province, China; 2 Department of Nursing, Beijing Ditan Hospital Capital Medical University, Beijing, China; 3 Beijing Home of Red Ribbon, Beijing Ditan Hospital Capital Medical University, Beijing, China; 4 Department of Quality Control, Beijing Ditan Hospital Capital Medical University, Beijing, China

**Keywords:** HIV/AIDS, Symptom distress, HIV duration, Older individual

## Abstract

**Background:**

Debate has persisted regarding whether PLWH with longer HIV durations have lower levels of prevalence and severity of symptoms compared with their newly diagnosed counterparts. Whether and how the HIV duration impact the symptom distress among middle-aged and older PLWH has not been explored clearly.

**Methods:**

The patients with HIV-infected aged more than 40 years old were included from seven designated medical institutions of seven regions in China. Outcome was the score of symptom distress. We used the multiple regression model to calculate adjusted Coefficient of Regression (β) with 95% CI in this study.

**Results:**

Among 210 participants (mean age 50.8 years; 71.0% male; 68.1% at asymptomatic stage) in the study, the median number of symptoms was 5. Of all symptoms reported, the most distressed symptoms were sleep disturbance (33.33%), followed by memory loss (31.90%), fatigue (26.67%), slow reactions (22.86%), and vision blur (21.90%). All participants were divided into four groups according to HIV duration, and the median of total score of symptom ditress among all participants was 0.1(0.0,0.9). Difference of total scores and clusters’ scores of symptom distress among four HIV duration groups were statistically significant. 51 participants with 6–10 years HIV duration were more likely to be higher level of education, at asymptomatic stage and have higher CD4 + T cell count. After adjustment for gender, age, race, education, marital status, employment, family income, region, stage of disease and CD4 + T cell count, the score of symptom distress among participants with 6–10 years HIV duration had was higher with the extension of HIV duration. Specially in physical symptoms and psychological symptoms,participants with 6–10 years HIV duration reported the persisting worse burdensome.

**Conclusions:**

HIV duration with 6–10 yearsmay be a key period that the medical team needs to pay special attention to among middle-aged and elderly PLWH. There is a need to provide medical and psychosocial services targeting middle-aged and elderly PLWH according to their changing symptom distress.

**Trial registration:**

Clinicaltrials.gov: ChiCTR2100046225. Registered 11 May 2021.

## Introduction

Despite major progress in the response, HIV epidemics continue to pose serious public health threats in all regions [1], having claimed 36.3 million [27.2–47.8 million] lives so far [2]. However, with increasing access to effective HIV prevention, diagnosis, treatment and care, HIV infection has become a manageable chronic health condition, enabling with people living with HIV (PLWH) achieving life expectancy approaching that of the general population [3]. This improvement in life expectancy and longevity has seen an increase in the number of PLWH living into older age [4]. In China, the highest HIV infection rate occurs in those aged between 20 and 40 years, but there has been an increasing prevalence in older adults recently, which is similar to trends in Western countries [5]. For example, the number of older PLWH has also increased rapidly in recent years in USA, more than 64% of people living with diagnosed HIV were aged 45–54 years (28%) and 55 years and older (35%) in 2018 [6]. A study in China showed that the ratio of younger cases to older cases decreased over time from 13.06 in 2005 to 3.05 in 2012 [7].The number of people aged 50 and above infected with HIV in China has been increasing, from 3.1 million in 2008 to 5.7 million in 2016. The proportion rose from 7.9% in 1990 to 15.6% in 2016 [7]. It can be said that the number of aging people infected with HIV or suffering from AIDS is increasing rapidly [8] and this elderly population has gradually become a high-risk group for AIDS Prevention and Control in China [9].

Due to gradually declining of the physical functions, the middle-aged and older PLWH differs from its younger counterparts in many ways [10], making them more likely to face comorbidities and need more complex medical care, facing more complex health and psychosocial challenges. With increasing age, comorbidities such as cardiovascular disease, diabetes, non-AIDS-defining cancers, and declines in renal and hepatic function become highly prevalent among PLWH [11–15]. Compared with individuals ageing without HIV infection, people ageing with HIV experience polypharmacy a decade earlier than uninfected individuals [4]. Despite further improvements in survival, PLWH are confronted with persistent symptoms resulting from all above health problems which impacts quality of life or well-being among PLWH [16]. Consequently, understanding and exploring on symptom experience of middle-aged and older PLWH is essential to further intervention and improve their health related quality of life.

Congruent with previous theories [17], symptom experience consisting of two different, linked concepts: symptom occurrence and symptom distress. Symptom distress reflects the emotional pathway and refers to the mental anguish or suffering caused by the symptom, having negative consequences on both physical and psychological health [18], which is meaningful and valuable. People’s ability to perceive distress from symptoms is thought to be influenced by various factors, including individual's characteristics, past experiences, society and culture, family role, and so on [19–24]. However, whether clinical stages or CD4 count affects the symptom distress among PLWH is still controversial. A study in New York twenty years ago among 504 ambulatory patients with AIDS demonstrated that neither gender nor CD4^+^ T-cell count was associated with symptom number or distress [25].Similar other studies also revealed that symptoms in HIV are present throughout the disease trajectory regardless of CD4^+^ T-cell count and stage classification and the burden of symptoms has not been carefully examined in terms of its association with clinical staging or treatment status [26, 27]. Especially in new ART era, some researchers from different countries conducted several cross-sectional surveys and found that prevalence and distress of symptoms were not associated with CD4^+^ T-cell count and clinical stage [28–30]. Nevertheless, some scientists found that pain was associated with immune status (CD4^+^ T-cell count) rather than other symptoms [31, 32], this is an interesting finding and need to be explored in the future. Additionally, an important study from McGowan reported the associations of age group with physical symptom distress and revealed that a longer time with diagnosed HIV infection, rather than age, is the dominating factor contributing to psychological morbidity and lower quality of life [29]. But other more scientists have been debating this may not be ture [33, 34]. As we known, symptoms often occur in clusters [35, 36]. How the different HIV duration impacts the different clusters of symptom distress among middle-aged and older PLWH has not been revealed clearly, which means more valuable for clinical practice than researches only focusing on single symptom. Moreover, a large number of previous studies collected data based on traditional universal scale not related to HIV symptoms, which may lead to bias or lack of disease specificity. Therefore, to address this knowledge gap, the aim of this study was to 1) explore the most burdensome symptom cluster in this population, 2) identify the association between symptom distress and different HIV duration.

## Methods

### Design

A multicenter, descriptive, cross-sectional design was used and this study was conducted in seven provinces or municipalities with different levels of incidence, including Xinjiang Province, Sichuan Province, Beijing, Guangdong Province, Qinghai Province, Henan Province and Liaoning Province. The investigation was conducted in the HIV/AIDS designated medical institutions of seven regions mentioned above, which are representative of eastern, southern, western and northern China.

### Sample

We recruited PLWH via convenience sampling from seven designated HIV/AIDS medical institutions in China from November 2021 to January 2022. PLWH were eligible for participation if they met the following inclusion criteria: (1) HIV infection diagnosed according to the Chinese AIDS Diagnosis and Treatment Guidelines (2021 edition), (2) more than 40 years old, (3) patients with antiretroviral therapy(ART) from inpatient and outpatient departments of seven designated medical institutions, and (4) informed consent to participate in this study. Excluded participants were those diagnosed with serious co-morbidities, cognitive impairment and those who were unable to complete the survey. We invited seven filed investigators who are in charge for recruiting participants and collecting data. We put up recruiting posters in seven designated HIV/AIDS medical institutions. Participants who were interested could ask filed investigator for some details. Before proceeding to the official survey, the potential participants were provided an online informed consent form which disclosed ethics issues in the study.

### Measures

Two instruments were used for this analysis.

1. Demographic Questionnaire A 21 item self-report sociodemographic questionnaire was used to collect demographic and illness background information. The demographic and socioeconomic variables included age, gender, ethnicity, education, marital status, employment status, economical income and residential district. The clinical variables were years since HIV diagnosis, years of ART, latest CD4 + T cell count, disease staging and comorbidities.

2. Self-report symptom scale A symptom checklist contains 27 items dividing into three domains (physical symptoms, cognitive symptoms, psychological symptoms), specific to the severity of HIV/AIDS-related symptoms [36], was used in this research. The content validity index for the whole checklist was 0.918. This checklist had good internal consistency, with a Cronbach’s alpha of 0.916 in this sample. In order to descript comprehensive overview of symptom experiences, we reviewed an extensive literature and merging the dimensions of the Memorial Symptom Assessment Scale (MSAS) [37, 38]into the self-report symptom scale(SRSS), which was one reliable and valid of instruments in previous survey. Participants were asked to indicate their responses by finishing a serials of steps. In the first step, participants would record if a symptom was experienced or not in past two weeks. If no, the participant would proceed to the next symptom. If the answer was yes, the participant was asked to characterize his or her experience concerning that particular symptom. The distress of symptoms rated by five-point Likert scale using the following options: 0 = “Not at all,” 1 = “A little bit,” 2 = “Somewhat,” 3 = “Quite a bit,” 4 = “Very much.” If a symptom is absent, each dimension is scored as 0 and the score for that symptom is 0. If a symptom is present, this symptom score is consistent with participants’ options. The scoring of the SRSS yields three symptom clusters’scores, including Physical Symptom Distress(PHSD), Cognitive Symptom Distress(COSD) and Psychological Symptom Distress (PSYSD), all of which add up to the total score. A higher score indicating a higher level of symptom distress.

### Data analysis

All the analyses were performed with the statistical software packages R (http://www.R-project.org, The R Foundation) and Free Statistics software versions 1.3. Descriptive analysis was applied to all participants. Categorical variables were expressed as proportions (%). Continuous data were expressed as mean and standard deviation(SD) or median and interquartile range (IQR), as appropriate. The statistical differences between HIV duration < 1 year, 1–5 years, 6–10 years and > 10 years were tested with the chi-square tests (categorical variables) and One-Way ANOVA (normal distribution), Kruskal–Wallis (skewed distribution) test, respectively. Coefficient of Regression(β) and 95% CIs were calculated for score of symptom distress with HIV duration using Multiple Linear Regression. We used unadjusted and multivariate adjusted models. In this study, the Multiple Linear Regression were adjusted for gender, age, race, education, marital status, annual family income, region, stage of disease and CD4 + T cell count. Tests for trend were conducted with linear regression by dividing HIV duration into four groups (< 1 years, 1–5 years, 6–10 years, > 10 years) as a variable in the models.

## Results

Among the 210 participants from the study, 149 male and 61 female were recruited. The mean age for the sample participants was 50.8 years (SD = 8.0). The participants were 89.5% (*n* = 188) Han, 10.5% (*n* = 22) Minority. Most participants, 52.4% (*n* = 110) had a lower school education and was married or cohabited. 83.3%(*n* = 175) participants’ family income were less than 6000 yuan per month. 68.1%(*n* = 143) of sample participants were diagnosed as being at asymptomatic stage. Participants with CD4^+^ T cell count of ≥ 500 cells/mm^3^ was 41.4% (*n* = 87). Participants with 6–10 years HIV duration were more likely to be higher level of education, at asymptomatic stage and have higher CD4^+^ T cell count. Difference of total scores and clusters’ scores of symptom distress among four HIV durations were statistically significant. Specially during 6–10 years after HIV-infected, the score of symptom distress was higher (see Table 1).

**Table 1:** Baseline characteristics of participants (*N* = 210)

Variables	HIV duration					
	Total (*n* = 210)	< 1 years (*n* = 28)	1–5 years (*n* = 85)	6–10 years (*n* = 51)	> 10 years (*n* = 46)	*p*-value
**Age, Mean ± SD**	50.8 ± 8.0	52.5 ± 8.8	49.7 ± 7.7	51.0 ± 6.9	51.6 ± 9.1	0.330
**Gender, n (%)**						0.581
female	61 (29.0)	7 (25.0)	24 (28.2)	13 (25.5)	17 (37.0)	
male	149 (71.0)	21 (75.0)	61 (71.8)	38 (74.5)	29 (63.0)	
**Race, n (%)**						0.078
Minority	22 (10.5)	0 (0)	10 (11.8)	4 (7.8)	8 (17.4)	
Han	188 (89.5)	28 (100)	75 (88.2)	47 (92.2)	38 (82.6)	
**Education, n (%)**						0.005
Middle school or below	110 (52.4)	20 (71.4)	46 (54.1)	18 (35.3)	26 (56.5)	
High school or equivalent	51 (24.3)	2 (7.1)	21 (24.7)	22 (43.1)	6 (13.0)	
Junior college or Undergraduate and above	49(23.4)	6 (21.4)	18 (21.2)	11 (21.6)	14 (30.4)	
**Marital status, n (%)**						0.277
Single	33 (15.7)	2 (7.1)	13 (15.3)	10 (19.6)	8 (17.4)	
Married or cohabiting	110 (52.4)	18 (64.3)	48 (56.5)	25 (49.0)	19 (41.3)	
Others	67 (31.9)	8 (28.6)	24 (28.3)	16 (31.4)	19 (41.3)	
**Employment, n (%)**						0.217
Employed	51 (24.3)	9 (32.1)	19 (22.4)	14 (27.5)	9 (19.6)	
Unemployed	33 (15.7)	6 (21.4)	8 (9.4)	11 (21.6)	8 (17.4)	
Freelancer	86 (41.0)	11 (39.3)	42 (49.4)	16 (31.4)	17 (37)	
Retired	40 (19.0)	2 (7.1)	16 (18.8)	10 (19.6)	12 (26.1)	
**Family income, n (%)**						0.746
< 3000 RMB	92 (43.8)	16 (57.1)	35 (41.2)	19 (37.3)	22 (47.8)	
3000–6000 RMB	83 (39.5)	9 (32.1)	36 (42.3)	22 (43.1)	16 (34.8)	
> 6000 RMB	35 (16.7)	3 (10.8)	14 (16.5)	10 (19.6)	8 (17.4)	
**Region, n (%)**						0.156
Urban	112 (53.3)	11 (39.3)	46 (54.1)	25 (49.0)	30 (65.2)	
Rural	98 (46.7)	17 (60.7)	39 (45.9)	26 (51.0)	16 (34.8)	
**Stage of disease, n (%)**						0.002
Acute stage	5 ( 2.4)	3 (10.7)	0 (0.0)	1 (2.0)	1 (2.2)	
Asymptomatic stage	143 (68.1)	15 (53.6)	62 (72.9)	42 (82.4)	24 (52.2)	
AIDS stage	62 (29.5)	10 (35.7)	23 (27.1)	8 (15.7)	21 (45.7)	
**CD4**^**+**^**T cell count, n (%)**						0.004
< 200	40 (19.0)	10 (35.7)	19 (22.4)	7 (13.7)	4 (8.7)	
200–499	83 (39.5)	15 (53.6)	33 (38.8)	19 (37.3)	16 (34.8)	
≥ 500	87 (41.4)	3 (10.7)	33 (38.8)	25 (49.0)	26 (56.5)	
**Comorbidities, n (%)**						0.143
No	171 (81.4)	23 (82.1)	73 (85.9)	36 (70.6)	39 (84.8)	
Yes	39 (18.6)	5 (17.9)	12 (14.1)	15 (29.4)	7 (15.2)	
PHSD, Median (IQR)	0.1 (0.0, 0.4)	0.1 (0.0, 0.3)	0.0 (0.0, 0.2)	0.3 (0.0, 0.8)	0.2 (0.0, 0.4)	< 0.001
COSD, Median (IQR)	0.0 (0.0, 0.4)	0.0 (0.0, 0.0)	0.0 (0.0, 0.2)	0.0 (0.0, 0.7)	0.0 (0.0, 0.6)	0.022
PSYSD, Median (IQR)	0.0 (0.0, 0.2)	0.0 (0.0, 0.2)	0.0 (0.0, 0.0)	0.0 (0.0, 0.5)	0.0 (0.0, 0.0)	0.010
Total.Score, Median (IQR)	0.1 (0.0, 0.9)	0.2 (0.0, 0.6)	0.0 (0.0, 0.5)	0.5 (0.0, 2.0)	0.3 (0.0, 1.2)	< 0.001

*PHSD* Physical Symptom Distress, *COSD* Cognitive Symptom Distress, *PSYSD* Psychological Symptom Distress

Table 2 reveals that the prevalence and distress of symptoms in this population. Of 210 participants, 34.2% reported no symptoms, while 5.7% reported only one symptom, 16.6% reported 2–3 symptoms, 10.4% reported 4–5 symptoms, 5.2% reported more than 6–7 symptoms, 7.0% reported 8–9 symptoms, and 20.5% reported more than 10 symptoms. The median number of symptoms was 5. Of all symptoms reported, the most distressed symptoms were sleep disturbance (33.33%), followed by memory loss (31.90%), fatigue (26.67%), slow reactions (22.86%), and vision blur (21.90%).

**Table 2:** Prevalence and Distress of Symptoms (*N* = 210)

Symptom	Number of Participants	Prevalence (%)	Distress (Mean ± SD)
Sleep disturbance	70	33.33	0.61 ± 1.03
Memory loss	67	31.90	0.59 ± 0.99
Fatigue	56	26.67	0.49 ± 0.99
Slow react	48	22.86	0.41 ± 0.89
Vision blur	46	21.90	0.40 ± 0.86
Low sex drive	43	20.48	0.41 ± 0.96
Hair loss	41	19.52	0.34 ± 0.84
Feeling down	39	18.57	0.30 ± 0.74
Having difficulty in concentrating	38	18.10	0.36 ± 0.90
Little interest in doing things	38	18.10	0.31 ± 0.77
Dizziness	37	17.62	0.32 ± 0.82
Muscle/joint ache	35	16.67	0.34 ± 0.89
Bloating/abdominal pain /diarrhea	35	16.67	0.29 ± 0.73
Hand/foot pain	34	16.19	0.31 ± 0.83
Feeling nervous	34	16.19	0.30 ± 0.76
Headache	33	15.71	0.28 ± 0.73
Lipodystrophy	32	15.24	0.24 ± 0.66
Uncontrollable worrying	30	14.29	0.27 ± 0.77
Fever	28	13.33	0.24 ± 0.69
Rash	28	13.33	0.26 ± 0.77
Having difficulty in reasoning	27	12.86	0.22 ± 0.66
Cough	25	11.90	0.19 ± 0.57
Weight loss	25	11.90	0.22 ± 0.71
Appetite loss	24	11.43	0.26 ± 0.83
Becoming confusing	23	10.95	0.20 ± 0.63
Mouth ulcer	19	9.05	0.12 ± 0.44
Nausea/vomit	15	7.14	0.14 ± 0.61

Table 3 presents the association between HIV duration and symptom distress. The coefficient (95% CI) of symptom distress was statistically significant for 6-10 years duration compared to the short and long HIV duration. In the non-adjusted model, participants who had 6-10 years HIV duration had a 0.803 times increasing of symptom distress (β = 0.803 [95% Cl 0.027, 1.579]). After adjustment for confounding factors in Table 1, the coefficients were 1.007 (0.189 ~ 1.825) and 1.155 (0.339 ~ 1.972) respectively (*p* < 0.001).

**Table 3:** Association between HIV duration and symptom distress in multiple regression model

Variable		Non-adjusted Coefficient (95% CI)	*P*-value	Model I Coefficient (95% CI)	*P*-value	Model II Coefficient(95% CI)	*P*-value
**HIV duration**	< 1 years	Ref		Ref		Ref	
	1–5 years	-0.094 (-0.813 ~ 0.625)	0.7983	0.161 (-0.586 ~ 0.909)	0.6728	0.202 (-0.553 ~ 0.956)	0.6011
	6–10 years	0.803 (0.027 ~ 1.579)	0.0437	1.007 (0.189 ~ 1.825)	0.0168	1.155 (0.339 ~ 1.972)	0.0061
	> 10 years	0.563 (-0.228 ~ 1.354)	0.1642	0.862 (0.033 ~ 1.692)	0.0430	0.755 (-0.087 ~ 1.597)	0.0806
**Age**		0.01 (-0.019 ~ 0.039)	0.4995	0.035 (-0.005 ~ 0.075)	0.0902	0.029 (-0.011 ~ 0.069)	0.153
**Gender**	Female	Ref		Ref		Ref	
	Male	-0.295 (-0.806 ~ 0.216)	0.2587	-0.09 (-0.662 ~ 0.483)	0.7585	-0.191 (-0.753 ~ 0.37)	0.5052
**Race**	Minority	Ref		Ref		Ref	
	Han	0.061 (-0.698 ~ 0.82)	0.8751	-0.194 (-1.015 ~ 0.627)	0.6439	-0.057 (-0.862 ~ 0.747)	0.889
**Education**	Middle school or below	Ref		Ref		Ref	
	High school or equivalent	0.11 (-0.463 ~ 0.683)	0.7072	0.566 (-0.074 ~ 1.206)	0.0846	0.622 (-0.014 ~ 1.258)	0.0567
	Junior college or Undergraduate and above	-0.099 (-0.708 ~ 0.51)	0.7504	0.56 (-0.175 ~ 1.294)	0.1372	0.569 (-0.151 ~ 1.288)	0.1232
	High school or equivalent	-0.011 (-1.429 ~ 1.408)	0.9883	1.265 (-0.489 ~ 3.019)	0.1592	1.144 (-0.594 ~ 2.883)	0.1986
**Marital status**	Single	Ref		Ref		Ref	
	Married or cohabiting	0.239 (-0.43 ~ 0.908)	0.4853	0.397 (-0.349 ~ 1.144)	0.2983	0.389 (-0.342 ~ 1.121)	0.2982
	Others	-0.224 (-0.975 ~ 0.526)	0.5585	-0.259 (-1.045 ~ 0.528)	0.5203	-0.232 (-1.015 ~ 0.55)	0.5617
**Employment**	Employed	Ref		Ref		Ref	Ref
	Unemployed	0.892 (0.147 ~ 1.637)	0.0199	0.498 (-0.407 ~ 1.404)	0.2819	0.288 (-0.629 ~ 1.204)	0.5391
	Freelancer	0.442 (-0.148 ~ 1.031)	0.1434	0.389 (-0.286 ~ 1.064)	0.2603	0.329 (-0.335 ~ 0.993)	0.3323
	Retired	0.14 (-0.564 ~ 0.845)	0.6969	-0.21 (-1.076 ~ 0.656)	0.6352	-0.273 (-1.121 ~ 0.574)	0.528
**Family income**	< 3000 RMB	Ref		Ref		Ref	Ref
	3000–6000 RMB	-0.791 (-1.292 ~ -0.291)	0.0022	-0.758 (-1.317 ~ -0.2)	0.0084	-0.725 (-1.276 ~ -0.173)	0.0109
	> 6000 RMB	-0.359 (-1.192 ~ 0.473)	0.3987	-0.753 (-1.7 ~ 0.193)	0.1205	-0.679 (-1.609 ~ 0.251)	0.1543
**Region**	Rural	Ref		Ref		Ref	Ref
	Urban	-0.431 (-0.894 ~ 0.031)	0.0691	-0.449 (-0.958 ~ 0.06)	0.0852	-0.49 (-0.994 ~ 0.015)	0.0587
**Stage of disease**	Acute stage	──	──	──	──	Ref	Ref
	Asymptomatic stage	──	──	──	──	-0.396 (-1.964 ~ 1.172)	0.6209
	AIDS stage	──	──	──	──	0.566 (-1.006 ~ 2.137)	0.4813
**CD4**^**+**^**T cell count**	< 200	──	──	──	──	Ref	Ref
	200–499	──	──	──	──	-0.162 (-0.815 ~ 0.492)	0.6283
	≥ 500	──	──	──	──	0.06 (-0.648 ~ 0.768)	0.8683

Model I: Adjust for gender, age, race, education, marital status, employment, family income, and region

Model II: Adjust for the variables in Model I plus stage of disease and CD4^+^T cell count

**Fig. 1:**
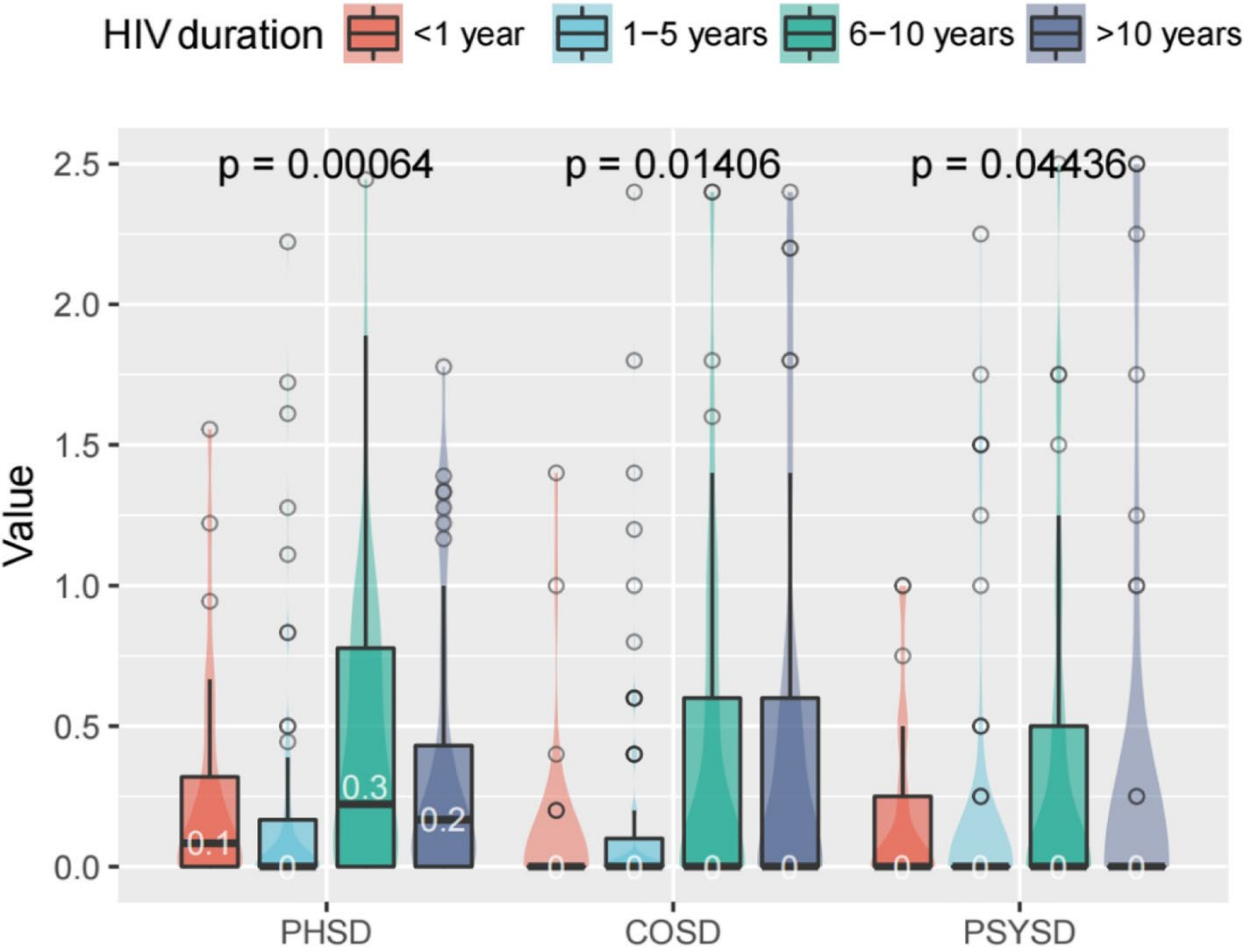
Differences in symptom distress among three clusters of symptoms. PHSD: Physical Symptom Distress. COSD: Cognitive Symptom Distress. PSYSD: Psychological Symptom Distress

**Fig. 2:**
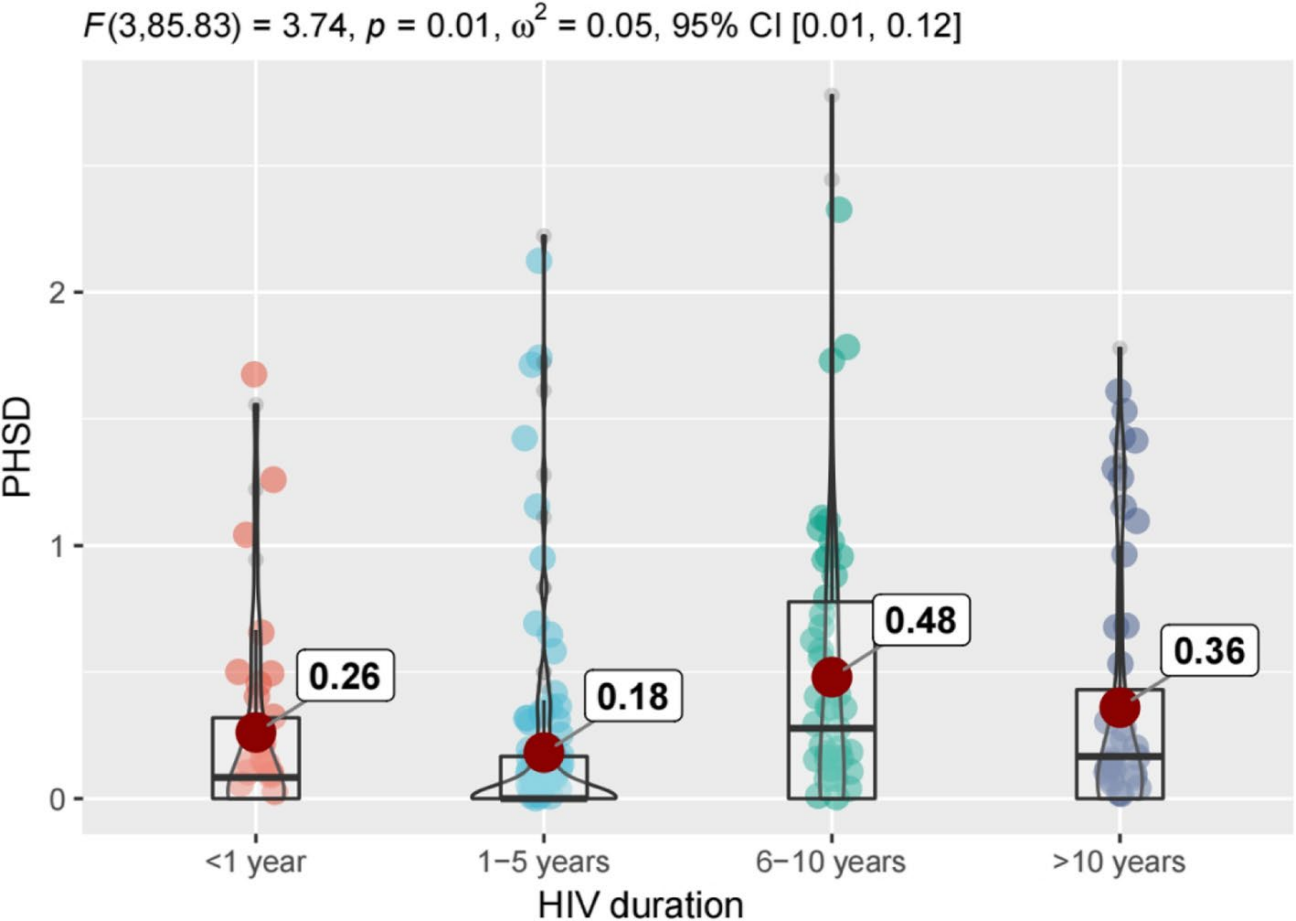
Differences of physical symptoms distress in different HIV duration. PHSD: Physical Symptom Distress

**Fig. 3:**
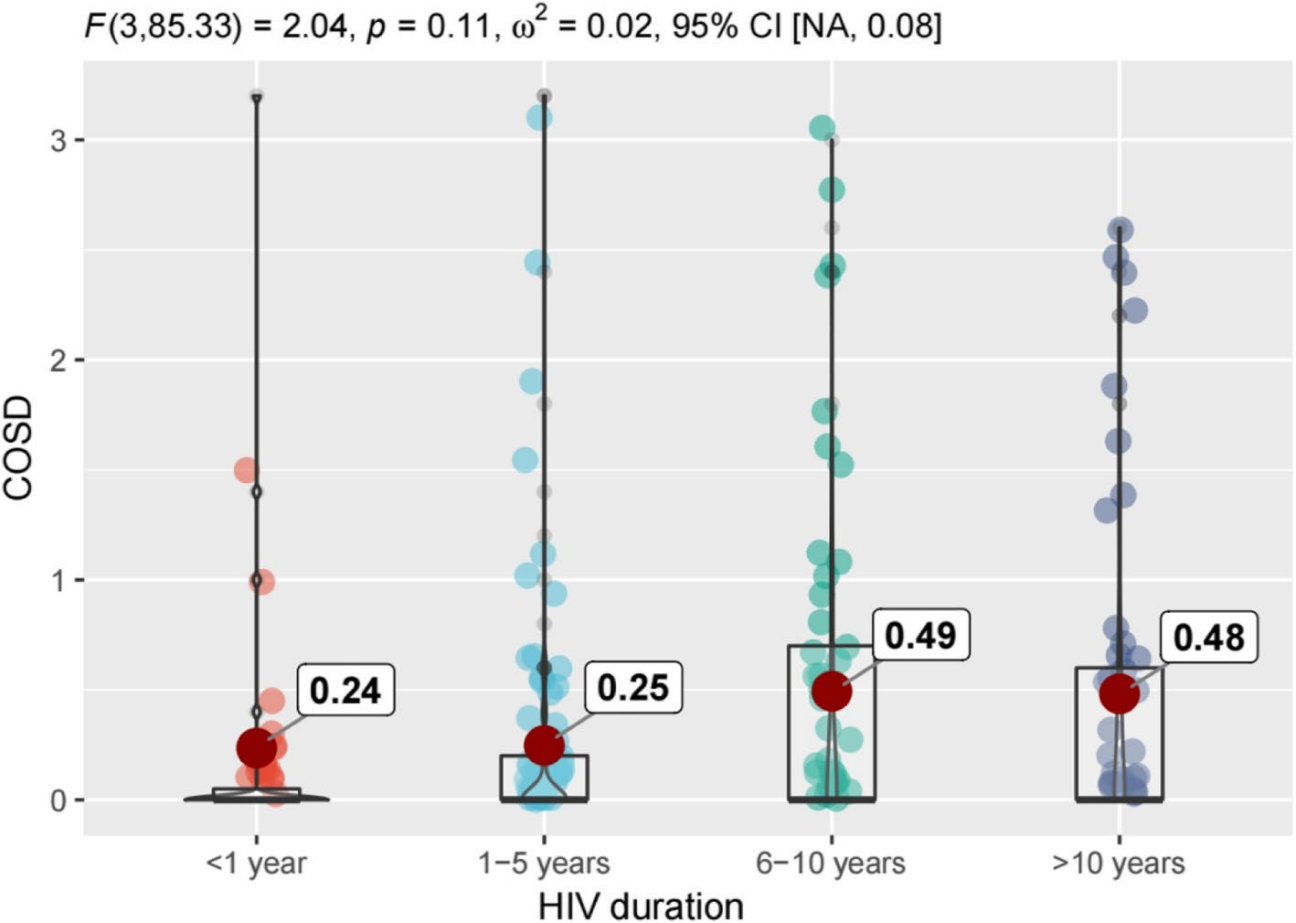
Differences of cognitive symptoms distress in different HIV duration. COSD: Cognitive Symptom Distress

**Fig. 4:**
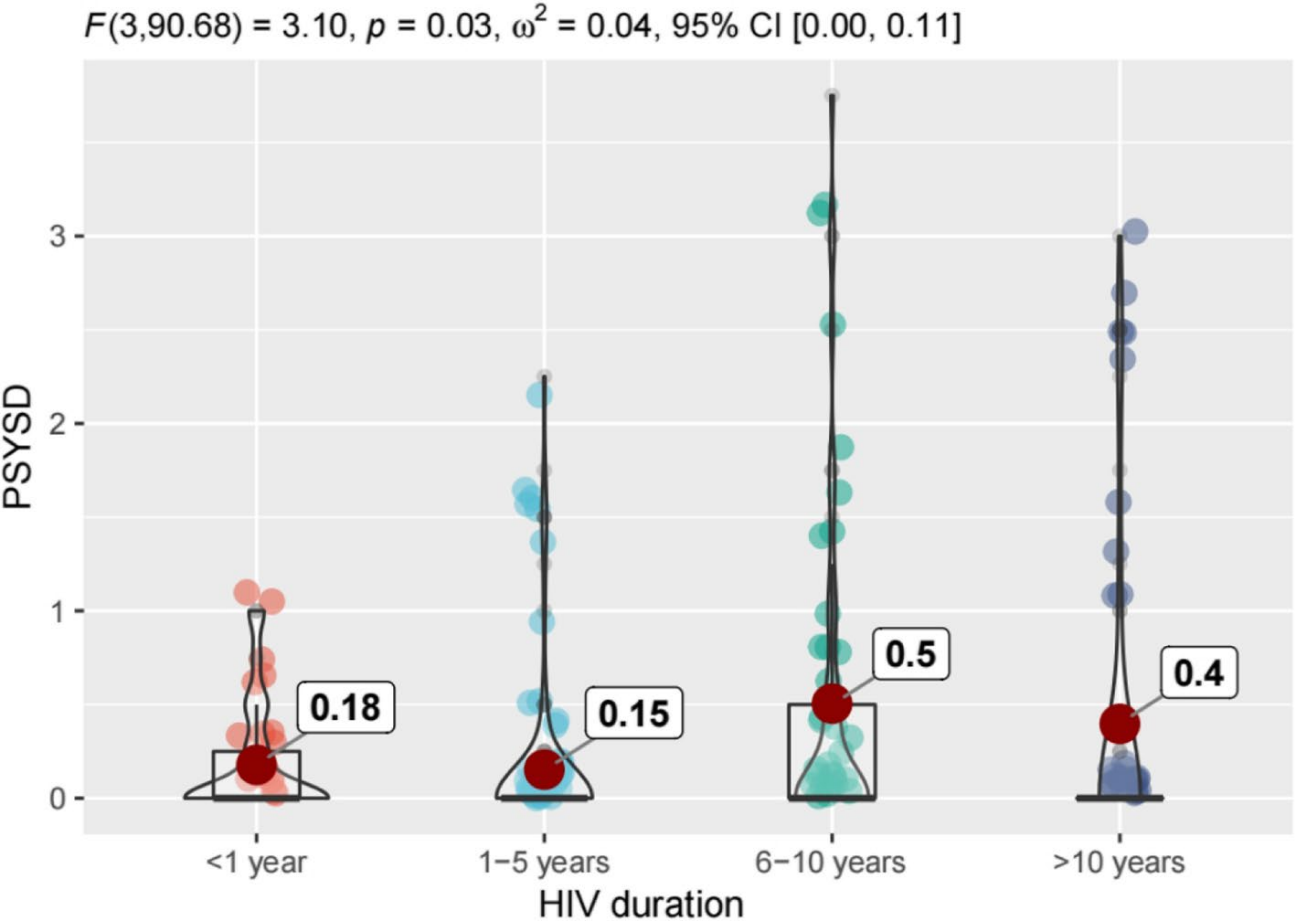
Differences of psychological symptoms distress in different HIV duration. PSYSD: Psychological Symptom Distress

Figures 1, 2, 3 and 4 shows that there are differences in symptom distress among three clusters of symptoms, including physical symptoms (fatigue, dizziness, headache, fever, cough, vision blur, sleep disturbance, rash, mouth ulcer, muscle/joint ache, hand/foot pain, appetite loss, bloating/abdominal pain /diarrhea, nausea/vomit, lipodystrophy, weight loss, low sex drive, hair loss),cognitive symptoms (having difficulty in concentrating, slow react, memory loss, having difficulty in reasoning, becoming confusing), psychological symptoms (uncontrollable worrying, feeling nervous, feeling down, little interest in doing things).In physical symptoms, participants with 6–10 years HIV duration reported the most distressed experience compared to other groups, while symptom distress decreased 10 years later. Nevertheless, in cognitive and psychological symptoms, those who were infected with HIV more than 6 years expressed the worse burdensome.

## Discussion

In this cross-sectional study, HIV duration was found to be independent associated with an increasing of the level of symptom distress. The relationship was characterized as follows: the symptom distress of middle-aged and older PLWH increased significantly during 6-10 years duration, but shorter or longer HIV duration does not increase the degree of distress. The burdensome feeling in three clusters demonstrated the same status, no matter for physical symptoms, cognitive symptoms and psychological symptoms, meaning that 6–10 years is key period for this population. A previous study demonstrated the similar evidence but not identical: longer HIV duration was strongly associated with a greater prevalence of functional problems, both overall and for each domain [29]. However, our outcome further highlighted that there were differences in symptom distress at different times. It suggested that we should pay more attention to the reason of increasing of symptom distress, assessment and management during 6-10 years duration. But this does not mean that other periods of symptom distress do not require attention and management.

We further revealed that distress of physical symptoms within one year after HIV-infected was worse than 1–5 years, due to sudden onset of many symptoms at acute stage or intolerability at the beginning of medication [39, 40]. Previous studies showed that following infection by HIV, approximately two thirds of patients have some symptoms attributable to an acute retroviral syndrome, and the most common manifestations of acute infection include fever up to 40C, malaise, anorexia or weight loss, myalgias, arthralgias, headache, diarrhea or oral, esophageal, and genital ulcers [41–43]. After ART treatment, the patient goes through a reaction phase after medication in the early stage, then the overall state of the patients gradually returns to stability [44, 45]. In our study, distress of physical and psychological symptoms of these population slightly decreased over a period of 1-5 years HIV duration, while cognitive symptoms remained unchanged. The distress of three clusters symptom demonstrated increasing over 6-10 years duration even continued to worsen in the following years, but for physical symptoms, the distressed feeling of middle-aged and older PLWH were relieved after 10 years of HIV infection. Our findings presented a phenomenon of inconsistent changes in symptom distress with the prolongation of HIV duration among different symptom clusters, and provided scientific reference for HIV care in the future.

Lampe et al. [46] revealed that physical and psychological symptoms were strongly predictive of viral rebound among patients on successful ART. Therefore, when HIV duration is more than 6 years or even longer, we should monitor and compare changes in symptom distress in order to capture patients’ clinical status rather than only depending on CD4^+^cell count. The cognitive impairment of elderly HIV patients is caused by HIV virus invasion, drug abuse, aging and other reasons [47].Therefore, our results also presented that continues increasing of cognitive complaints as the extension of time of HIV invasion. Additionally, some studies have found concordance between cognitive complaints and an individual’s performance on neuropsychological testing [48–50], and Wilkie et al. [51] argued that “poor neuropsychological test performance prior to the development of AIDS is a proximal predictor of mortality”, which suggested that cognitive impairment may be indicate future clinical outcome and health-related quality of life. Despite the concealment and slow development of cognitive symptoms, medical workers should not neglect the evaluation and intervention from the early stage. Early identification of cognitive impairment can result in appropriate clinical interventions in remediable conditions and in the improvement of quality of life [47].

A study conducted in UK reported the most prevalent distressing physical symptoms were: lack of energy/tiredness (26%), difficulty sleeping (24%), muscle-ache/joint pain (21%) and pain (18%) in similar population [29, 30]. But we got completely different results: some symptoms with a high prevalence of distress in our sample in this study were similar to those common elderly people without HIV infection, which showed the advances in ART medication in the new era and less side effects. However, we found a lower prevalence of distressing symptoms in the over 40 s group compared with younger age groups, which is accordance in a previous study [29]. This could, in part, be attributable to older adults attributing health changes to natural ageing and therefore not rating them as distressing [52, 53].

### Limitations

This study is the first to explore the association between distress of different symptom clusters and HIV duration among middle-aged and older PLWH in China. However, there are still some limitations. This study was based on a cross-sectional sample. Therefore, it is just an exploration research rather than deriving accurate causality. All symptom data were from self-report and based only participants may overestimate or underestimate their really conditions. Meanwhile, the number or type of symptoms reported may be limited due to 27 items scale, despite this assessment tool has been validated in Chinese PLWH. Additionally, HIV infection duration was collected in the form of classified variable in order to ensure accurate and efficient data collection in seven regions of China, which slightly restricted the in-depth exploration of association. Future studies should address this issue.

## Conclusions

As the HIV epidemic spreads in middle aged and older people, greater attention is being directed to the importance of symptom recognition and management in this population. Longer time with diagnosed HIV infection, however, was related to a higher prevalence of all self-rated health problems, for example, symptom distress, independently of age. Especially in 6–10 years duration, the symptoms distress among middle-aged and older PLWH may reach the peak, therefore it is more necessary to strengthen the assessment and management. Distress of three symptom clusters presents different status during the different HIV durations and suggests that there is a need to provide medical and psychosocial services targeting elderly PLWH according to their changing symptom distress. A greater likelihood to report sleep disturbance, memory loss, fatigue, slow reactions, and vision blur and a lower likelihood to report headaches, dizziness, mouth ulcer, or diarrhea among older patients may be helpful in the future management of patients [54].

## Data Availability

The datasets generated and/or analysed during the current study are not publicly available due to the privacy from people living with HIV but are available from the corresponding author on reasonable request.
